# Pelvic Exenteration: Experience from a Rural Cancer Center in Developing World

**DOI:** 10.1155/2015/729658

**Published:** 2015-02-08

**Authors:** Sampada B. Dessai, Satheesan Balasubramanian, Vijay M. Patil, Santam Chakraborty, Atanu Bhattacharjee, Syam Vikram

**Affiliations:** ^1^Department of Surgical Oncology, Malabar Cancer Center, Thalassery-Koppalam-Panoor Road, Moozhikkara, Kodiyeri, Thalassery, Kannur, Kerala 670103, India; ^2^Department of Clinical Hematology and Medical Oncology, Malabar Cancer Center, Thalassery-Koppalam-Panoor Road, Moozhikkara, Kodiyeri, Thalassery, Kannur, Kerala 670103, India; ^3^Department of Radiation Oncology, Malabar Cancer Center, Thalassery-Koppalam-Panoor Road, Moozhikkara, Kodiyeri, Thalassery, Kannur, Kerala 670103, India; ^4^Division of Clinical Research and Biostatistics, Malabar Cancer Center, Thalassery-Koppalam-Panoor Road, Moozhikkara, Kodiyeri, Thalassery, Kannur, Kerala 670103, India

## Abstract

*Background*. Pelvic exenteration (PE) is a morbid procedure. Ours is a rural based cancer center limited trained surgical oncology staff. Hence, this audit was planned to evaluate morbidity and outcomes of all patients undergoing PE at our center. *Methods*. This is a IRB approved retrospective audit of all patients who underwent PE at our center from January 2010 to August 2013. The toxicity grades were retrospectively assigned according to the CTCAE version 4.02 criteria. Chi-square test was done to identify factors affecting grades 3–5 morbidity. Kaplan Meier survival analysis has been used for estimation of median PFS and OS. *Results*. 34 patients were identified, with the median age of 52 years (28–73 years). Total, anterior, posterior, and modified posterior exenterations were performed in 4 (11.8%), 5 (14.7%), 14 (41.2%), and 11 (32.4%) patients, respectively. The median time for surgery was 5.5 hours (3–8 hours). The median blood loss was 500 mL (200–4000 mL). CTCAE version 4.02 grades 3-4 toxicity was seen in nine patients (25.7%). The median estimated progression free survival was 31.76 months (25.13–38.40 months). The 2-year overall survival was 97.14%. *Conclusion*. PE related grades 3–5 morbidity of 25.7% and mortality of 2.9% at our resource limited center are encouraging.

## 1. Introduction

Pelvic exenteration (PE) was first described in 1948 by Brunschwig as a form of radical surgery for recurrent carcinoma cervix [1]. As originally described, total pelvic exenteration entails en masse removal of all pelvic viscera followed by end colostomy and bilateral ureteric implantation in the colon above the stoma [1]. Several authors have described the use of this surgery in the management of recurrent and advanced neoplasms of the bladder, rectum, and other gynecological malignancies [2–5].

PE is an extensive surgery [6], with reported perioperative mortality rate as high as 23% [1]. However, with improvement in surgical techniques and postoperative care, a decrease in mortality has been noted in newer series [7]. Though world literature has many reports from western world about the effectiveness of pelvic exenteration, very few reports are from India [6, 8]. This is surprising as most patients with pelvic malignancies in India present with locally advanced stages [9] and may be a related to the shortage of experienced surgical oncologists in India [10].

Outcome in surgical oncology depends on the skill of the surgeon as well as the volume of surgery performed [11]. Studies have shown that outcomes for the patients with abdominal malignancies were improved when operated upon in a high volume center [12]. Ours is a rural based cancer center in the public sector with a steadily increasing patient load. In addition, trained surgical oncologists were available only from 2009 onwards. Hence, this audit was planned to evaluate morbidity and outcomes of all patients undergoing pelvic exenteration at our center over three years.

## 2. Material and Methods

This is an institutional review board approved retrospective audit of all patients who underwent PE at our center from January 2010 to August 2013. Patients were identified from operation theatre registers maintained during the above-mentioned time period. Data are obtained on demographic profile, primary tumor site, previous treatment, indication for PE, intraoperative and postoperative complications, adjuvant treatment, and failure. The toxicity grades were retrospectively assigned according to the Common Terminology Criteria for Adverse Events (CTCAE) version 4.02 (National Cancer Institute) criteria.

The types of pelvic exenteration were classified as below [13, 14]:

1. total pelvic exenteration: hysterectomy, cystectomy, low anterior resection or APR, +/− sigmoid resection, and removal of pelvic lymph nodes;
2. anterior pelvic exenteration with ileal conduit (Brookes technique): hysterectomy, cystectomy, and removal of pelvic lymph nodes;
3. posterior pelvic exenteration: hysterectomy with abdominoperineal resection;
4. modified posterior pelvic exenteration: hysterectomy with low anterior resection and coloanal anastomosis.

The postoperative care differed according to the site of tumor and according to the type of surgery. However, in general patients were kept in surgical ICU for 48 hours, they were mobilized on the second day, antibiotics were provided for 5–7 days, and deep venous thrombosis prophylaxis was provided till discharge. Oral feeds were started on second or third day depending upon the presence of stoma and/or bowel movements. Prior to this partial parenteral nutrition was provided under the guidance of the dietetics division. Stoma/ileal conduit care was provided by the specialized nursing unit along with preoperative and postoperative counselling.

During their indoor stay (calculated from day of surgery till discharge) patients were monitored as follows:

1. hemodynamic monitoring: D0 till discharge,
2. monitoring for arterial pH: D0-D1,
3. complete hemogram, serum electrolytes, and renal function tests: performed daily from D0 till patients started full diet and required no more IV fluids.

Descriptive statistics reported include frequencies for categorical variables and median (range) for continuous variables. Chi-square test was done to identify factors associated with grades 3–5 morbidity. Kaplan Meier survival analysis was used for estimation of median progression free survival (PFS) and overall survival (OS). PFS was calculated from the date of surgery till date of progression or death, whichever was earlier. Patients were censored at the last date of follow-up if still not progressed. OS was calculated from the date of surgery till date of death, with patients being censored at the last date of follow-up if still alive. SPSS version 16 was used for analysis.

## 3. Results

### 3.1. Demographic Pattern

**Figure 1:**
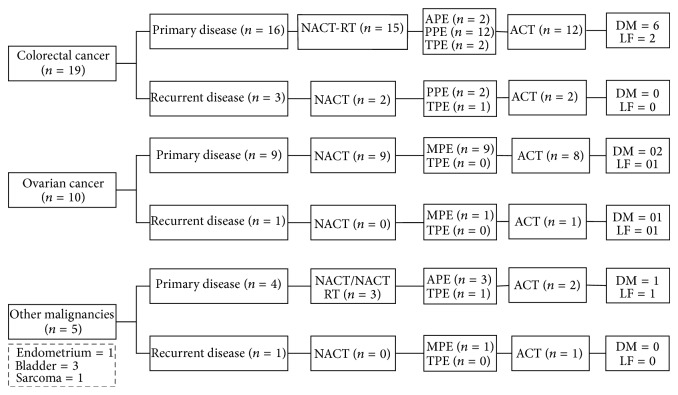
Consort diagram of patients. NACT: neoadjuvant chemotherapy, NACT-RT: neoadjuvant chemoradiation, APE: anterior pelvic exenteration, PPE: posterior pelvic exenteration, MPE: modified pelvic exenteration, TPE: total pelvic exenteration, ACT: adjuvant chemotherapy, LF: local failure, and DF: distant failure.

Thirty-four patients were identified subjected to the above-mentioned criteria.  Figure 1 provides the consort diagram. The median age was 52 years (28–73 years). There were 28 females and 6 male patients. All patients had ECOG PS 0-1. Nine patients had hypertension and six patients had diabetes mellitus, all of which were medically controlled. In twenty-nine patients the exenteration was done as a part of the primary treatment, while in five patients it was done for recurrent disease. In the 5 patients with recurrence, the median disease free interval was 14 months (range 9–48 months) prior to PE.

### 3.2. Tumor Details and Indication of Exenteration

The details of primary tumor and indication of exenteration are shown in Table 1. The primary site of tumor was in rectum or sigmoid in 19 patients, in ovary or endometrium in 11 patients, in bladder in 3 patients, and in retroperitoneum in one patient. The indications for performing a PE were locally advanced tumor extending beyond the organ of origin in 29 patients and recurrence limited to pelvis in 5 patients. In 15 patients with rectal cancer preoperative chemoradiation was given. In nine patients with upfront ovarian cancer neoadjuvant chemotherapy (NACT) was given using three weekly paclitaxel and carboplatin.

### 3.3. Surgical Details

Total, anterior, posterior, and modified posterior exenterations were performed in 4 (11.8%), 5 (14.7%), 14 (41.2%), and 11 (32.4%) patients, respectively. Intraoperatively in 7 patients bladder was involved, in 4 patients disease was extending up to the pelvic side walls, and in 2 patients sacrum was involved. All patients had R0 resection. Sacral resection was done in 2 patients and accompanying lymph node dissection was done in 33 patients. In the patient with retroperitoneal sarcoma lymph node dissection was not done. The details of lymph node dissection done are given in Table 2. The median time for surgery was 5.5 hours (3–8 hours). The median blood loss was 500 mL (200–4000 mL).

### 3.4. Complications

The median interval of indoor admission was 11 days (6–32 days). CTCAE version 4.02 grades 3-4 toxicity was seen in nine patients (25.7%). One patient succumbed to postoperative sepsis on the 12th postoperative day. Postoperative ICU admissions were required in all patients. The details of other toxicity are shown in Table 3.

There was no difference in grades 3–5 morbidity according to age and type of exenteration or according to the disease status (primary or recurrent). Though the rate of grades 3–5 morbidity was 36.66% in patients with age 60 years and above, as opposed to 20.8% in patients below the age of 60 years, it was not statistically significant. (*P* = 0.329). The factor which affected grades 3–5 morbidity was site of primary. In patients with nonrectal primary the incidence of grades 3–5 morbidity was 11.1% versus 41.2% in patients having a rectal primary (*P* = 0.042).

Details of other toxicities are shown in Table 3. There was no difference in grades 3–5 morbidity according to age and type of exenteration or according to the disease status (primary or recurrent). Though the rate of grades 3–5 morbidity was 36.66% in patients with age 60 years and above, as opposed to 20.8% in patients below the age of 60 years, it was not statistically significant (*P* = 0.329). The factor which affected grades 3–5 morbidity was site of primary. In patients with nonrectal primary the incidence of grades 3–5 morbidity was 11.1% as compared to 41.2% in patients having a rectal primary (*P* = 0.042).

### 3.5. Adjuvant Treatment

Adjuvant treatment was received in 28 patients. Adjuvant chemotherapy was delivered in 26 patients while adjuvant radiation was given in 2 patients, both of whom had rectal cancers.

Among ovarian cancer patients, as noted above, 9 patients had received NACT expect one rest all 8 completed adjuvant chemotherapy. They all received total 6 cycles of paclitaxel and carboplatin. One patient of ovarian cancer developed a myocardial infarction postoperatively with a decline in ejection fraction which precluded further adjuvant treatment. One patient with ovarian cancer who underwent exenteration for recurrent disease received 6 cycles of adjuvant gemcitabine and carboplatin regimen.

In 20 patients with colorectal cancer, 17 patients received capecitabine oxaliplatin regimen for 8 cycles, while 3 patients declined further adjuvant chemotherapy. All these 3 patients had rectal cancers, one of whom had received upfront chemoradiation. Adjuvant pelvic radiation was delivered to the other 2 patients to a total dose of 50.4 Gy in 28 fractions.

### 3.6. Failure Pattern

At a mean follow-up of 18 months, 10 patients had progressed. The predominant site of failure was systemic in 8 patients. Three patients had local failure only, while 2 patients had local with systemic failure.

### 3.7. Survival

The median estimated progression free survival was 31.76 months (25.13–38.40 months). The median overall survival was not reached. Only one patient had died. The 2-year overall survival was 97.14%. Outcomes according to different sites of tumor are depicted in Table 4.

## 4. Discussion

Pelvic exenteration is an important procedure indicated in various gynecological, gastrointestinal, and other pelvic tumors [3, 5, 15]. Traditionally this procedure is associated with high mortality and morbidity. Mortality rates as high as 23% were described in mid-twentieth century with a mean time of mortality of 8 days [1]. Subsequently, however, refinements in surgical techniques with better supportive care facilities have decreased the mortality associated with this procedure to below 5–10% [7, 16–18]. However, still considerable proportion of patients (23–44%) have severe morbidity associated with this procedure [7, 16–18]. In our series, the rate of grades 3–5 toxicity was 25.7% and postoperative mortality within 30 days was 2.9%. These figures are in concurrence with major series reported in the twenty first century [16–22].

Ours is a rural based cancer center with limited manpower. From 2010 to 2013, only 2 trained surgical oncologists were working at this center catering to more than 2000 patients each per year. In addition, there was only one full time anesthetist working during this period at the center. The institute was devoid of a critical care specialist until recently which may be the underlying reason behind majority of grades 3–5 adverse events being biochemical and hematological disturbances. In spite of these shortcomings, the overall results of morbidity and mortality are encouraging.

The institute started doing these surgeries from 2010. It is known that, in oncological surgeries, high volumes and standardization of techniques lead to better surgical outcomes [11, 12]. Selected experiences reported on pelvic exenteration surgeries from other centers show that on average 2-3 pelvic exenterative surgeries are done per year [7, 19–22]. At our center on average we do nearly 10 PE per year. Moreover, there has been a steady increase in these surgeries from 3 exenterations been done in 2010 to 15 done till August in the year 2013.

The reasons for this low mortality and morbidity are just not limited to the high volume and surgical experience at our centre. The selection criteria for such surgeries need to be stringent. In our series, all patients had ECOG PS0-1. None of these patients had uncontrolled comorbidities. The median age was near 50 years. This means that fairly young patients with good performance status were selected. In addition, most of the surgeries were performed in the primary setting which is known to be associated with reduced morbidity as compared to surgery in the setting of recurrent disease [19, 23, 24].

Posterior exenteration or modified posterior exenteration was done in majority of patients in this series, as majority of the patients had colorectal primaries. Modified posterior exenteration procedure has same amount of morbidity as a cytoreductive surgery at least in ovarian cancers [25]. All patients had received prophylactic antibiotics, DVT prophylaxis, and intensive nursing care in the postoperative period. All these factors taken together may have contributed to the low mortality and morbidity in this series [7]. The single mortality seen in this series was that of a young patient who had undergone total pelvic exenteration for a recurrence of rectal cancer. She had sepsis with blood culture positive for colistin resistant* Acinetobacter baumannii* and succumbed to the infection on the 12th postoperative day.

Our rate of surgical R0 resection compares favourably with other series reporting an R0 resection rate of 46.3%–68.4% which have been reported [20, 26–28]. Thus, decision regarding operability in such patients should be based on a combination of clinical and radiological findings. However, R1 and R2 pelvic exenteration resections are known to be associated with poor prognosis and should be avoided [20, 26–28].

The predominantly distant failure pattern seen in the present series is intriguing, as majority of the patients had rectal cancers and received systemic chemotherapy. However, such a predominant systemic mode of failure has also been reported in the literature in both rectal and gynecological primaries treated with pelvic exenteration [29, 30].

The limitations of the present study were a mixed patient population, modest sample size, limited follow-up, and a retrospective design. However, these surgeries were performed in a two-year time period without significant heterogeneity in treatment policies. In order to minimize bias we have reported outcomes of all patients undergoing PE during the time period. However, a median PFS of nearly 32 months is a promising finding especially in light of the resource limitations. The two-year overall survival achieved is 97.14% which is in line with outcomes reported for patients undergoing primary PE in modern series [6, 20, 27, 31, 32].

## 5. Conclusion

PE related grades 3–5 morbidity of 25.7% and mortality of 2.9% at our resource limited center are encouraging. Further improvements in outcomes are expected with increased availability of critical care expertise. These results should encourage the uptake of this treatment modality in other centres in resource limited nations with a higher burden of advanced pelvic malignancies.

## Figures and Tables

**Table 1:** Cross tabulation of tumor site and indication for exenteration.

Tumor site	Locally advanced tumor beyond organ of origin	Recurrence limited to pelvic organs
Rectum/rectosigmoid	16 patients	3 patients
Ovary	9 patients	1 patient
Endometrium	00	1 patient
Retroperitoneal sarcoma	01 patient	00
Bladder	03	00

**Table 2:** Details of lymph node dissection performed.

Variable	Value
Lymph node dissection done	33 patients
Type of LN dissection	
Pelvic + paraaortic	11 patients
Pelvic	21 patients
Pericolic	01 patient
Median number of LN retrieved	12 nodes (1–39 nodes)

**Table 3:** Acute toxicity within 30 days of surgery.

	Grade 1	Grade 2	Grade 3	Grade 4	Grade 5
Intraoperative bowel/ureter/venous injury	0	0	0	0	0
Wound infection	1	4	2	0	0
Colonic fistula	0	0	0	0	0
Small intestinal obstruction	0	0	0	0	0
Ureteric anastomotic leak	0	0	0	0	0
Postoperative hemorrhage	0	0	1	0	0
Ventricular arrhythmia	1	0	0	0	0
Skin ulceration^*^	0	0	0	0	0
Rise in serum creatinine	0	2	0	0	0
Metabolic acidosis	1	0	1	0	0
Sepsis	2	0	0	0	1
Hyponatremia	16	NA	6	0	0
Hypernatremia	3	1	0	0	0
Hypokalemia	13	7	1	0	0
Hyperkalemia	0	1	1	0	0
Hypomagnesemia	0	0	0	0	0
Anemia	16	10	4	0	0
Thromboembolic event^**^	0	1	0	0	0
Myocardial infarction	0	0	0	1	0

Numbers depicted are numbers of patients. ^*^Ulceration of skin due to pressure ulcer. ^**^The event was deep venous thrombosis.

**Table 4:** Table depicting outcomes (overall survival and progression free survival) in major sites.

	*N*	Two-year PFS (95% CI)	Two-year OS
Colorectal cancer	20	62.5% (39–100%)	94.7% (85–100%)
Ovarian cancer	10	37.5% (08–100%)	100%
